# Total utilization of lignin and carbohydrates in *Eucalyptus grandis*: an integrated biorefinery strategy towards phenolics, levulinic acid, and furfural

**DOI:** 10.1186/s13068-019-1644-z

**Published:** 2020-01-06

**Authors:** Xue Chen, Kaili Zhang, Ling-Ping Xiao, Run-Cang Sun, Guoyong Song

**Affiliations:** 1 Beijing Key Laboratory of Lignocellulosic Chemistry, Beijing Forestry University, Beijing, 100083 China; 2 Center for Lignocellulose Science and Engineering, Liaoning Key Laboratory of Pulp and Paper Engineering, School of Light Industry and Chemical Engineering, Dalian Polytechnic University, Dalian, 116034 China

**Keywords:** *Eucalyptus*, Reductive catalytic fractionation, Phenolic monomers, Platform chemicals

## Abstract

**Background:**

Lignocellulosic biomass, which is composed of cellulose, hemicellulose and lignin, represents the most abundant renewable carbon source with significant potential for the production of sustainable chemicals and fuels. Current biorefineries focus on cellulose and hemicellulose valorization, whereas lignin is treated as a waste product and is burned to supply energy to the biorefineries. The depolymerization of lignin into well-defined mono-aromatic chemicals suitable for downstream processing is recognized increasingly as an important starting point for lignin valorization. In this study, conversion of all three components of *Eucalyptus grandis* into the corresponding monomeric chemicals was investigated using solid and acidic catalyst in sequence.

**Results:**

Lignin was depolymerized into well-defined monomeric phenols in the first step using a Pd/C catalyst. The maximum phenolic monomers yield of 49.8 wt% was achieved at 240 °C for 4 h under 30 atm H_2_. In the monomers, 4-propanol guaiacol (12.9 wt%) and 4-propanol syringol (31.9 wt%) were identified as the two major phenolic products with 90% selectivity. High retention of cellulose and hemicellulose pulp was also obtained, which was treated with FeCl_3_ catalyst to attain 5-hydroxymethylfurfural, levulinic acid and furfural simultaneously. The optimal reaction condition for the co-conversion of hemicellulose and cellulose was established as 190 °C and 100 min, from which furfural and levulinic acid were obtained in 55.9% and 73.6% yields, respectively. Ultimately, 54% of *Eucalyptus* sawdust can be converted into well-defined chemicals under such an integrated biorefinery method.

**Conclusions:**

A two-step process (reductive catalytic fractionation followed by FeCl_3_ catalysis) allows the fractionation of all the three biopolymers (cellulose, hemicellulose, and lignin) in *Eucalyptus* biomass, which provides a promising strategy to make high-value chemicals from sustainable biomass.
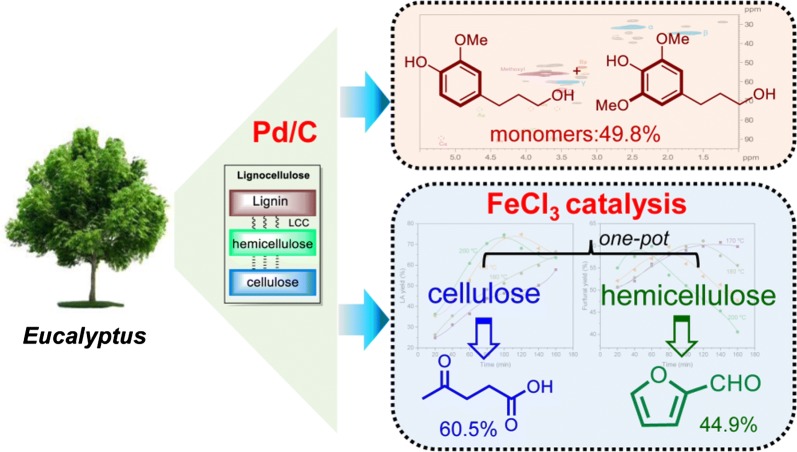

## Background

Lignocellulosic biomass, which is composed of lignin, cellulose, and hemicellulose, represents the prevalent sustainable hydrocarbon resource for impactful conversion into our future chemicals and fuels [1–3]. Achieving high overall product yields from all three biopolymers in an integrated process has great potential to promote future biorefinery technologies. Current lignocellulosic biorefinery predominantly gears to carbohydrate (cellulose and hemicellulose) valorization [4–6]. Typical biorefinery procedures included a pretreatment process, i.e., Kraft, sulphite and organosolv processes, by which lignin component was initially extracted and separated [7–9]. Alternatively, direct acid-treatment of biomass sawdust was also established, wherein (hemi)cellulose components were preferentially converted into platform compounds, such as furfural, 5-hydroxymethylfurfural (5-HMF), levulinic acid (LA), and so on [10–14]. Of note, these procedures negatively affect the depolymerization of lignin into monomers because stable C–C linkages would be formed irreversibly through depolymerization–recondensation of lignin [15, 16]. As a consequence, the upgrading of lignin to useful products has not been realized in a successful version.

The depolymerization of lignin into well-defined mono-aromatic chemicals suitable for downstream processing would be an important starting point for the valorization of lignin as well as the promotion of the economics of biorefineries [17, 18]. Recently, the direct reductive catalytic fractionation (RCF) of lignocellulosic biomass was emerged as a new protocol for biomass fractionation and lignin depolymerization [15, 19]. Such processes usually employed a heterogeneous catalyst based on ruthenium [20, 21], palladium [22–28], nickel [29–31], copper [32], and molybdenum metals [33–35]. During RCF, protolignin in lignocellulose matrix was firstly depolymerized into phenolic monomers through the cleavage of C–O bonds in β-*O*-4 linkages [8, 35]. The selectivity-control of monophenols bearing different endchains at *para* position, such as propyl [22, 36], propenyl [37], propanol [38], ethyl and allyl ether [34, 35], has been realized by the choice of catalysts and/or additives [39]. The RCF process also left a solid fraction as a pulp containing cellulose and hemicellulose components with high retentions, which could be readily retrieved by filtration process. This provides an ideal carbohydrate feedstock for subsequent upgrading because the biomass recalcitrance has been broken down. For most cases, the cellulose component could be readily enzymatically hydrolyzed to glucose after RCF process [23, 40, 41]. Hydrolytic hydrogenation of (hemi)cellulose residues into pentitols from hemicellulose and hexitols from cellulose has already been studied by using Ru/C catalyst [20].

The hydrolysis of either hemicellulose into furfural, or cellulose into 5-HMF (and subsequent LA) has been widely reported [42–44], while integrated catalytic conversion of this two biopolymers to co-produce platform compounds is still limited, because of the significant difference in their chemical properties [45]. Dumesic and coworkers reported that the hemicellulose and cellulose components in corn stover sawdust can be converted into levulinic acid and furfural simultaneously with H_2_SO_4_ catalyst in γ-valerolactone (GVL) [11]. Wyman et al. found that metal halides could serve as an efficient catalyst for the production of furfural, 5-HMF and LA with high yields from lignocellulosic biomass [46]. A recent work by Abu-Omar and coworkers suggested that the solid hemi(cellulose) residue, which generated from Ni-catalyzed RCF of Miscanthus, could be converted into LA and furfural with a Lewis acid catalyst under microwave heating [47].

**Fig. 1:**
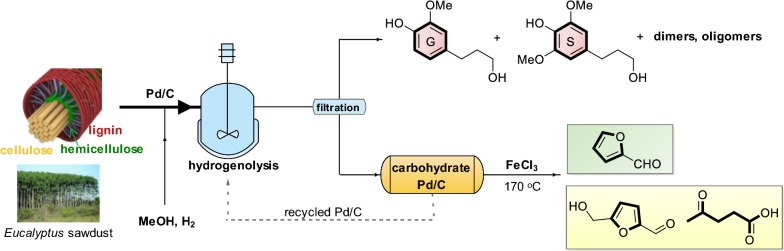
Schematic representation of the reductive catalytic fractionation (RCF) of *Eucalyptus*

*Eucalyptus* is considered as one of the fastest-growing sources of fiber, which is widely applied to artificial board, pulping, and paper-making industries owing to its wide adaptability and high photosynthesis. We recent reported the RCF of hardwood, softwood and herbaceous plants with Mo [33–35], Ni [30, 31] and Pd [23] catalysts. These results encourage us to continually explore the transformation of all three biopolymers into monomeric chemicals based on RCF process. In this paper, we used Pd/C as a catalyst to treat *Eucalyptus* sawdust, thus resulting in 4-propanol guaiacol/syringol with high yield from depolymerization of lignin component. High retention of cellulose and hemicellulose pulp was also obtained, which on the treatment with cheaply available FeCl_3_ catalyst led to the formation of 5-HMF, LA and furfural simultaneously. Finally, all the three biopolymers (cellulose, hemicellulose, and lignin) in *Eucalyptus* biomass were efficiently transformed into well-defined chemicals and fuels via an integrated protocol involving RCF and acidic catalysis processes (Fig. 1).

## Results and discussion

### Monomeric phenols from RCF of *Eucalyptus*

**Fig. 2:**
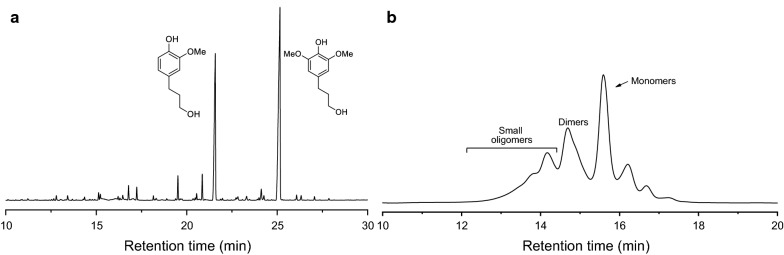
Gas chromatogram (**a**) and molecular weight distribution (**b**) of lignin oil generated from hydrogenolysis of *Eucalyptus* sawdust at 240 °C for 4 h

A series of heterogeneous metal catalysts have been employed in direct treatment of biomass sawdust. In view of the combination of high yield of phenolic monomers and high retention of carbohydrate pulp, Pd/C was chosen as the catalyst [23]. The RCF reaction of *Eucalyptus* sawdust (40–60 mesh) was carried out in a batch reactor with Pd/C (10 wt% dosage) under H_2_ (30 atm) in methanol. A range of temperatures were screened for 4 h. The lignin monomer yields, the average molecular weight (*M*_w_) of the lignin oil and the retention of the sugars are summarized in Table 1 (Additional file 1: Table S2). The soluble fraction contained monomeric, dimeric and oligomeric phenols derived from depolymerization of lignin, as well as polyols derived from partial hydrolysis of (hemi)cellulose. The identification and quantification of lignin monomers were assessed by GC–MS, by comparison with corresponding authentic samples. A typical monomer distribution is illustrated in Fig. 2a (Additional file 1: Table S2). Increased total phenolic monomer yields were observed with the rise of reaction temperature in a range of 200–240 °C, thus reaching maximum yield of 49.8 wt%. Commonly, the maximum theoretical yield of monomers will be approximately equal to the square of the fraction of cleavable β-*O*-4 structures in the lignin polymer structure [48]. In view of ca. 70% β-*O*-4 linkages have been quantified in *Eucalyptus* protolignin [49], the here obtained monomers yield has reached the theoretic maximum value. In the monomers, 4-propanol guaiacol (12.9 wt%) and 4-propanol syringol (31.9 wt%) were identified as the two major phenolic products with 90% selectivity, being consistent with previous report [38]. A proposed pathway to the major products included the cleavage of C–O bonds in β-*O*-4 linkages leading to monolignols and subsequent reduction of C=C bonds, and both steps would be catalyzed by Pd/C. The RCF reaction at 200 °C for 8 h and 250 °C for 2 h with Pd/C catalyst was also performed (Additional file 1: Table S3, entries 5–6), which afforded phenolic monomer yields in 36.6 and 30.9 wt%, respectively. Several solvents were screened in the RCF of *Eucalyptus sawdust* in presence of Pd/C under 240 °C, 4 h and H_2_ atmosphere (Additional file 1: Table S3, entries 1–4). Ethanol, isopropanol and water gave 42.4, 40.0 and 35.0 wt% yields of monomeric phenols, which were lower than that in MeOH (49.8 wt%). The high polarity of methanol is conducive to the extraction of lignin due to its permeation into the lignocellulosic matrix and the conversion of lignin oligomers to phenolic monomers. Finally, temperature for 240 °C and time for 4 h in MeOH were chosen as the optimized condition for RCF reaction of *Eucalyptus*. Gel permeation chromatography (GPC) analysis exhibited a significant decrease in molecular weight (*M*_w_ = 470–430 g mol^−1^) relative to the isolated milled wood lignin (MWL, 5630 g/mol) [50] and enzymatic mild acidolysis lignin (EMAL, 8100 g mol^−1^) from *Eucalyptus* tree [34].

**Table 1:** Comparison of the results after the RCF of *Eucalyptus* under different temperatures

Entry	Temp. (°C)	*M*_W_ (g mol^−1^)	Phenolic monomers yield (wt%)^a^				Sugar retention (wt%)^b^	
					Others	Total	C6	C5
1	200	470	7.3	18.0	1.1	26.4	81.7	87.9
2	220	460	8.2	19.8	2.6	30.6	82.6	82.9
3	230	460	10.8	26.8	4.1	41.7	81.5	72.3
4	240	450	12.9	31.9	5.0	49.8	82.5	67.8
5	250	430	11.8	28.8	4.1	44.7	68.8	57.2

Reaction conditions: *Eucalyptus* (1.0 g), 10 wt% Pd/C catalyst (100 mg), MeOH (40 mL), H_2_ (30 atm), 4 h

^a^Based on total lignin content in *Eucalyptus*

^b^Based on the chemical composition of insoluble fractions

**Fig. 3:**
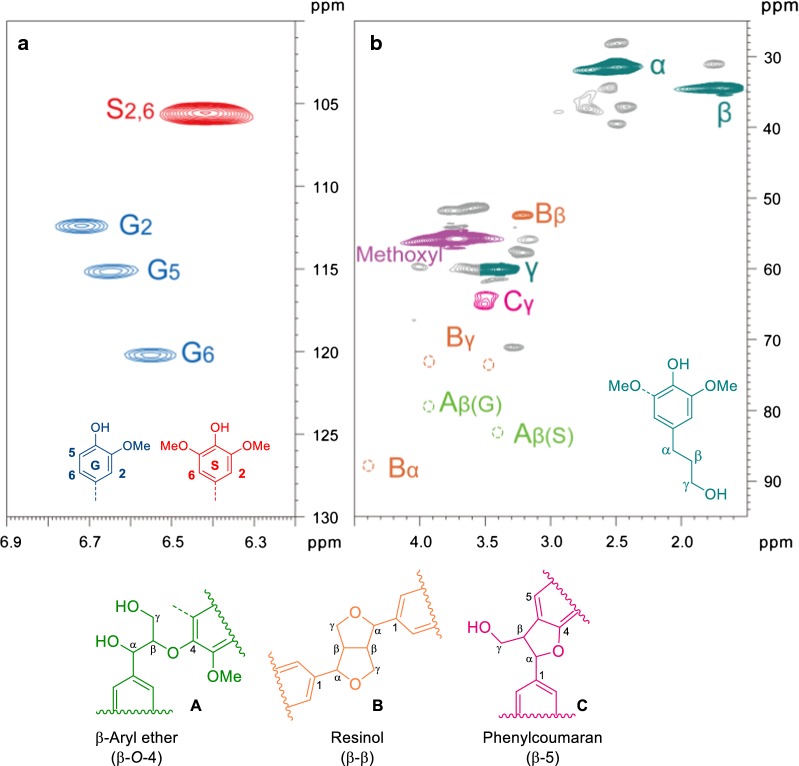
2D-HSQC NMR spectra of the lignin oil obtained from 240 °C, 4 h (DMSO-*d*_6_). **a** Aromatic region. The signals for G and S were found at 112.2/6.71 (G_2_), 115.0/6.64 (G_5_), 120.0/6.55 (G_6_) and 105.4/6.41 (S_2,6_) ppm, respectively. **b** Side chain region. The signals for propanol moiety were found at 31.7/2.49, 34.5/1.68 and 60.1/3.40 ppm, respectively

A 2D HSQC NMR spectra of the lignin oil was collected (Fig. 3). The main substructures, such as β-aryl ether (β-*O*-4, A), resinol (β–β, B), phenyl coumaran (β-5, C), have disappeared after Pd/C treatment, indicating a nearly thorough C–O bonds dissociation in the protolignin. Newly, dominant cross peaks which correspond to a propanol moiety (31.7/2.49, 34.5/1.68 and 60.1/3.40 ppm, labeled in atrovirens) were observed in HSQC, in line with the observation of GC–MS analysis. The cross signals at aromatic region corresponding to guaiacyl (G) and syringyl (S) were also found at 112.2/6.71 (G_2_), 115.0/6.64 (G_5_), 120.0/6.55 (G_6_) and 105.4/6.41 ppm (S_2,6_), respectively.

**Fig. 4:**
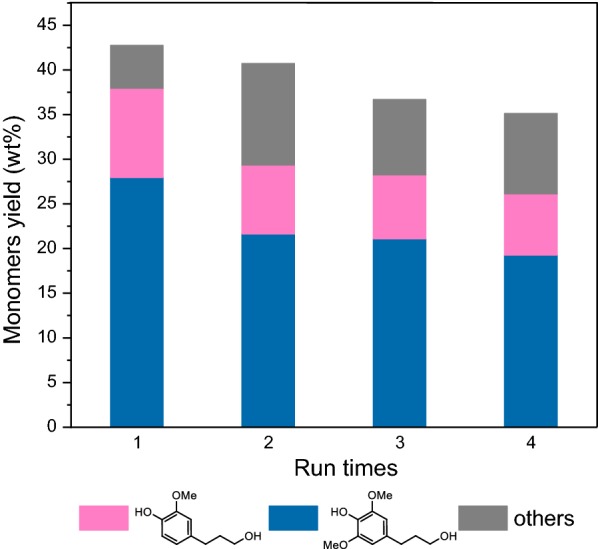
Results of recycling the Pd/C for *Eucalyptus* sawdust

The carbohydrate and the catalyst were left in the insoluble fraction after RCF treatment, which could be separated from the reaction mixture through filtration. Screening of the solid residue with a 300-mesh sieve can recover 91% of Pd/C, which was then subjected to simple washing and was used directly in the following cycle. As shown in Fig. 4, the yields of phenolic monomers were still retained at 35.1 wt% in the fourth run, with high selectivity towards 4-propanol guaiacol and 4-propanol syringol. These results indicated a good recycling stability of the Pd/C catalyst, which is important for the potential applications in industry.

### Conversion of the solid carbohydrate residue to 5-HMF, LA and furfural

The chemical composition of leftover carbohydrate pulp, obtained at the maximum phenolic monomers concurrently, was analyzed by NREL procedure. The retentions of cellulose and hemicellulose in carbohydrate pulp were determined as 82.5 wt% and 67.8 wt%, respectively. XRD-analysis (Additional file 1: Fig. S4) showed that the crystallinity of the carbohydrate pulp (60.2%) was much higher than that of the *Eucalyptus* sawdust (36.2%), mainly due to the removal of lignin component from biomass matrix and the degradation of amorphous cellulose under current reaction condition. The degree of delignification was also determined as 80.6%, suggesting that the lignin carbohydrate complexes have been destroyed and the solid carbohydrate pulp is suitable candidate for the co-production of platform chemicals.

**Fig. 5:**
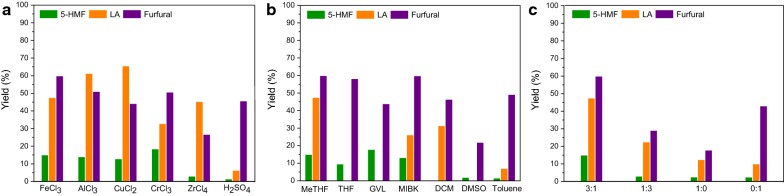
Conversion of solid carbohydrate residue to platform chemicals. **a** Catalysts screening in MeTHF/saturated NaCl (3:1) aqueous solution; **b** different organic solvent with saturated NaCl aqueous solution in 3:1 ratio; **c** ratios screening of MeTHF and saturated NaCl aqueous solution. General conditions: carbohydrate pulp (0.15 g), metal halide catalysts in biphasic solvent (0.1 M, 12 mL), 170 °C for 120 min

In an acidic reaction condition, the transformation of hemicellulose into furfural is much sooner than the generation of 5-HMF and levulinic acid from cellulose [51]. Of note, the competing side reactions of (hemi)cellulose leading to unwanted humins are also involved in acid-catalysis systems. To find the proper balance among the efficiency and selectivity of these two biopolymers conversion, as well as avoiding the condensation, is important in maximizing the co-production of platform chemicals in an integrated process. Initially, the conversion of carbohydrate pulp was performed in a biphasic solvent system composed by 2-methyl tetrahydrofuran (MeTHF) and saturated NaCl aqueous solution with a volume ratio of 3:1. A series of metal chloride catalysts, such as FeCl_3_, AlCl_3_, CuCl_2_·2H_2_O, CrCl_3_ and ZrCl_4_, were screened at 170 °C for 120 min (Fig. 5a). High conversions of cellulose were observed, from which 5-HMF and levulinic acid were both generated in high combined yields (48.3–78.4%). Obviously, levulinic acid was the primary product, probably because 5-HMF is readily hydrolyzed into levulinic acid and formic acid under current acidic conditions [52]. This scenario was in line with the observations of acid-treated biomass sawdust [53]. For the case of hemicellulose, FeCl_3_ was superior to other metal chlorides, which gave furfural in 59.9% yield. A strong acid, H_2_SO_4_ was also utilized to treat the carbohydrate pulp, which outperformed in the conversion of hemicellulose fraction into furfural (45.7%). In consideration of total products from carbohydrate pulp, FeCl_3_ was chosen as the best catalyst for the further investigation.

Reaction medium is a critical factor determining the reaction environment in the transformation of biomass feedstocks to platform chemicals [54]. To study the role of the solvent, some organic solvents combined with saturated NaCl aqueous solution in 3:1 ratio were tested in the presence of FeCl_3_ at 170 °C for 120 min (Fig. 5b). Apart from dimethyl sulfoxide (DMSO), furfural could be generated from hemicellulose in a desirable fashion in different biphasic systems (43.9–59.9%). Significant influence on cellulose conversion was observed with the variation of organic solvent partner. Apparently, DMSO or toluene is not a proper organic integrant, in which poor detectable products derived from cellulose were generated. When THF or GVL was combined with NaCl aqueous solution, only 5-HMF was formed, with no observation of levulinic acid. This scenario is consistent with the observation from reaction of cellulose [55, 56] and biomass sawdust [46, 57]. On the contrary, levulinic acid (31.4%) was detected exclusively when dichloromethane (DCM) was partnered. In the case of MeTHF and methylisobutylketone (MIBK), levulinic acid was generated as the main product in 47.6% and 26.1% yields, respectively, together with the observation of 5-HMF. Thereby, the ingredient of organic solvent in biphasic systems can modulate the reactivity and selectivity of carbohydrate transformation. The influence of volume ratio of MeTHF and saturated NaCl aqueous solution was also investigated, as shown in Fig. 5c. Varying the MeTHF–water ratio from 3:1 to 1:3 led to the decrease of furfural (29.1%), 5-HMF (3.0%) and levulinic acid (22.5%) synchronously. A sole solvent, such as MeTHF or saturated NaCl aqueous solution was used, in which reduced yields of 5-HMF and levulinic acid were observed. Obviously, biphasic solvent plays an imperative role in promoting the conversion of cellulose by the partitioning of resulted 5-HMF and levulinic acid into organic MeTHF phase [58]. Finally, the biphasic system composed with MeTHF and saturated NaCl aqueous solution in 3:1 ratio proved to be the best choice for the co-conversion of cellulose and hemicellulose.

**Fig. 6:**
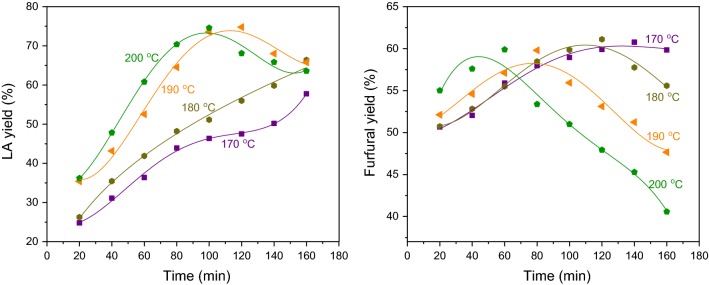
Effect of reaction temperature and time on solid carbohydrate residue conversion to furfural and LA. Reaction conditions: carbohydrate pulp (0.15 g), FeCl_3_ in MeTHF/saturated NaCl aqueous solution (0.1 M, 12 mL)

To assess the influence of reaction time and temperature on the co-production platform compounds, reaction time-course data at different temperatures were obtained and shown in Fig. 6. Either raising reaction temperature or prolonging reaction time enhanced the production of levulinic acid, because harsh condition is favorable to the depolymerization of the crystalline cellulose and subsequent hydrolysis reaction. The temperatures of 170 °C and 180 °C were not sufficient to form levulinic acid entirely, and maximal yield (74.6%) of levulinic acid was achieved at 200 °C, 100 min or 190 °C, 120 min. Under such a case, no 5-HMF was observed, suggesting a complete conversion from 5-HMF to levulinic acid. Further prolonging reaction time at 190 °C or 200 °C resulted in the decrease of levulinic acid, probably because of the formation of humin.

For the case of conversion of hemicellulose, the formation of furfural was increasing smoothly with the reaction time at a relative low temperature (such as 170 °C), and the yield reached to 60.8% in 140 min finally. This confirmed that the conversion of hemicellulose occurred more readily than cellulose. At higher temperatures (180 °C, 190 °C and 200 °C), the maximum yield of furfural (ca. 60%) could be realized rapidly; however, dramatic decline of furfural was also observed with prolonging the reaction time, because high temperatures not only facilitated the dehydration of xylose, but also accelerated the degradation of furfural [59]. Based on the above experiment results, the optimal reaction condition for the co-conversion hemicellulose and cellulose was established as 190 °C and 100 min, from which furfural and levulinic acid were obtained in 55.9% and 73.6% yield, respectively.

### A mass balance of the RCF combined with FeCl_3_ catalysis

**Fig. 7:**
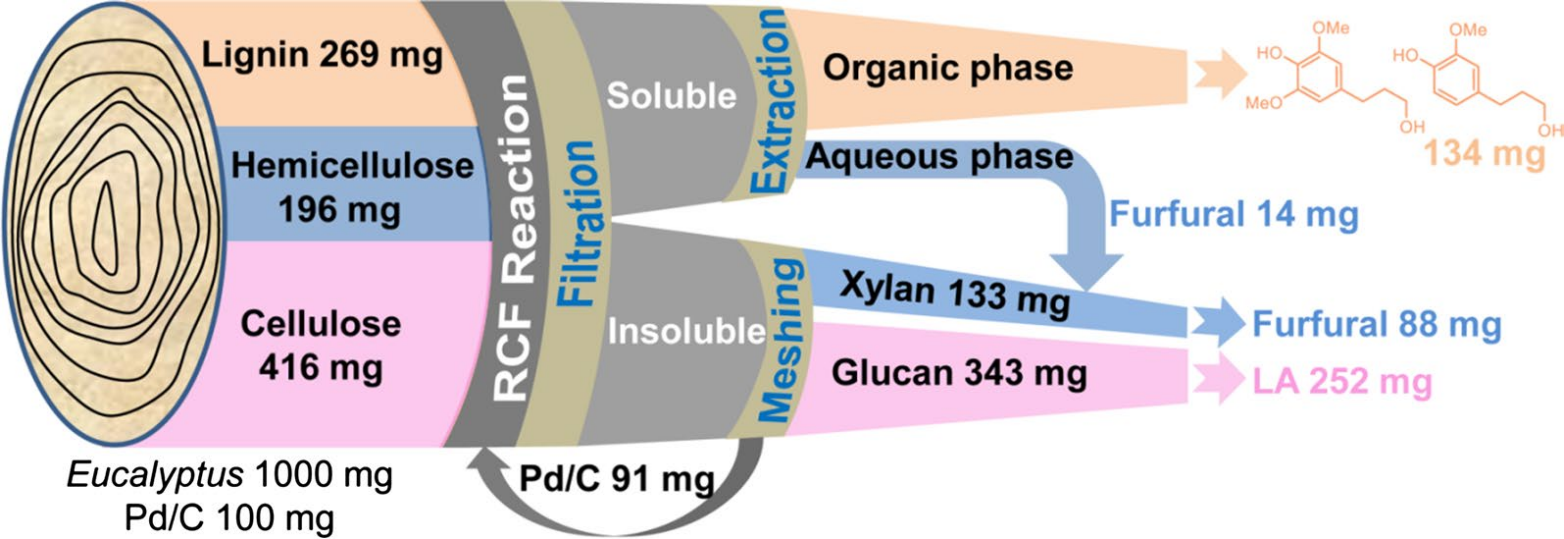
Mass balance calculation of the reductive catalytic fractionation (RCF) combined with FeCl_3_ catalysis

A process mass balance of the combination of RCF with FeCl_3_ catalysis was described based on 1.0 g of dried *Eucalyptus* (Fig. 7). Under optimized conditions, lignin component (ca. 269 mg) in biomass matrix can be depolymerized into monomers (134 mg), dimers and oligomers through RCF process, which could be solubilized in methanol. Under such a condition, the leftover solid residue, contained cellulose (343 mg), hemicellulose (133 mg) and Pd/C catalyst. Upon the treatment of FeCl_3_, the leftover carbohydrate pulp yielded furfural (74 mg) and levulinic acid (252 mg) in an integrated version. Partial xylan presented in methanol, could also be recovered and transferred into furfural (14 mg). The resulted products, i.e., levulinic acid, furfural and monophenols, corresponded to 61% of cellulose, 45% of hemicellulose and 50% of lignin, respectively. Ultimately, 54% of *Eucalyptus* sawdust can be converted into well-defined chemicals under such an integrated biorefinery method.

## Conclusions

RCF followed by FeCl_3_ catalysis was employed for the fractionation of all three components in *Eucalyptus*. Results showed that high monomers yield of 49.8 wt% close to the theoretical maximum was obtained during RCF process, next to high retentions of cellulose and hemicellulose components. Then, FeCl_3_ was used to convert the cellulose and hemicellulose in carbohydrate pulp to 5-HMF, LA and furfural simultaneously. The furfural and LA yields reached 55.9% and 73.6%, respectively, when the reaction condition was established as 190 °C and 100 min. This study provides an efficient method for fractionation and sequential utilization of all three major components in *Eucalyptus*.

## Materials and methods

### Material

*Eucalyptus* sawdust was harvested from Guangxi Province. The *Eucalyptus* sawdust was mainly composed of 41.6% cellulose, 19.6% hemicellulose, 27.0% lignin, 3.2% extract, and 0.2% ash. The dried *Eucalyptus* was grounded in a mill to obtain a 40–60 mesh fraction, which was further extracted with toluene/ethanol (2:1, v/v) and dried for further experiment. Pd/C catalyst (where Pd content is 5 wt%) was purchased from Energy Chemical. The metal halide catalysts (FeCl_3_, AlCl_3_, CuCl_2_·2H_2_O, CrCl_3_, and ZrCl_4_) were used and loaded based on their equivalent anhydrous mass to achieve 0.10 M catalyst loading.

### General catalytic reaction

RCF experiments were performed in a 100-mL stainless steel batch reactor (Parr instruments Co.) equipped with a 60-mL glass tube. In a typical reaction, extracted *Eucalyptus* sawdust (1.0 g), Pd/C (100 mg, 10 wt%) and methanol (40 mL) were charged into the reactor, which was then sealed, flushed with N_2_ and pressurized with H_2_ at room temperature. The reaction mixture was heated at desired temperature with magnetic stirring (800 rpm). After reaction, the reactor was cooled immediately by ice water. The mixture was filtered with Buchner funnel, thus giving soluble fraction containing phenolic compounds and insoluble fractions containing carbohydrate pulp and Pd/C.

### Lignin products analysis

The soluble fraction was concentrated by a rotary evaporation to remove methanol and extracted with dichloromethane and water. Subsequent removal of dichloromethane of the organic phase gave a brown “lignin oil”. The lignin oil with a standard (tetradecane) was dissolved in dichloromethane in a 10-mL volumetric flask for determination, which was analyed by gas chromatography coupled with mass spectrometer (GC–MS) and gas chromatography (GC) to identify and quantify the monomeric phenols as described in Additional file 1. The phenolic monomers yield and sugar retention were calculated from equations:

$${\text{Phenolic}}\;{\text{monomers }}\;{\text{yield }}\left( {{\text{wt}}\% } \right) = \frac{{m\left( {{\text{total}}\;{\text{phenolic}}\;{\text{monomers}}} \right)}}{{m\left( {{\text{total}}\;{\text{lignin}}} \right)}} \times 100\% ,$$

$${\text{C}}6\;{\text{sugar}}\;{\text{retention }}\left( {{\text{wt}}\% } \right) = \frac{{m\left( {{\text{cellulose}}\;{\text{in}}\;{\text{insoluble}}\;{\text{fraction}}} \right)}}{{m\left( {{\text{total}}\;{\text{cellulose}}} \right)}} \times 100\% ,$$

$${\text{C}}5\;{\text{sugar}}\;{\text{retention }}\left( {{\text{wt}}\% } \right) = \frac{{m\left( {{\text{hemicellulose}}\;{\text{in}}\;{\text{insoluble}}\;{\text{fraction}}} \right)}}{{m\left( {{\text{total}}\;{\text{hemicellulose}}} \right)}} \times 100\% .$$

The molecular weight of the lignin oil was determined by gel permeation chromatography (GPC) equipped with a UV detector (254 nm) and PL-gel mixed-B column (10 mm × 7.5 mm i.d.), where THF was as the solvent (0.5 mL/min). A THF solution of lignin oil (2 mg/mL) was used for injection. Two-dimensional heteronuclear single quantum coherence (2D HSQC) NMR analysis was acquired on a Bruker Avance 400 MHz spectrometer using DMSO-*d*_6_ (0.5 mL) as a solvent [60].

### Compositional analysis of carbohydrate pulp

The Pd/C catalyst in the insoluble fraction could be separated and recycled in 91% yield through a mesh screening (300 mesh). The remaining carbohydrate pulp was then subjected to chemical composition analysis by sulfuric acid hydrolysis according to the National Renewable Energy Laboratory (NREL) procedure [61]. The chemical composition of the *Eucalyptus* sawdust was also determined by NREL procedure.

### Conversion of the carbohydrate pulp into LA and furfural

Typically, the experiments were carried out in a 15-mL sealed tube with carbohydrate pulp (150 mg), catalyst (0.10 M) and solvent (12 mL) under magnetic stirring. The tube in stainless steel reactor was heated to a given temperature (170, 180, 190, and 200 °C) and maintained for different times (20, 40, 60, 80, 100, 120, 140, and 160 min). After the reaction, the reactant was cooled to room temperature and extracted with dichloromethane and water, and the organic phase was subjected to high-performance liquid chromatography (HPLC) analysis. The detailed analytical method is presented in Additional file 1. LA and FF yields were calculated based on following equations:

$${\text{LA}}\;{\text{yield }}\left( \% \right) = \frac{{{\text{moles}}\;{\text{of}}\;{\text{LA}}\;{\text{produced}}}}{{{\text{moles}}\;{\text{of}}\;{\text{staring}}\;{\text{glucan}}\;{\text{in}}\;{\text{carbohydrate}}\;{\text{pulp}}}} \times 100\% ,$$

$${\text{FF}}\;{\text{yield }}\left( \% \right) = \frac{{{\text{moles}}\;{\text{of}}\;{\text{FF}}\;{\text{produced}}}}{{{\text{moles}}\;{\text{of}}\;{\text{staring}}\;{\text{xylan}}\;{\text{in}}\;{\text{carbohydrate}}\;{\text{pulp}}}} \times 100\% .$$

## Supplementary information

**Additional file 1: Table S1.** The chemical composition of *Eucalyptus* sawdust and carbohydrate pulp. **Table S2.** Reductive catalytic fractionation of *Eucalyptus* under different temperature. **Table S3.** Reductive catalytic fractionation of *Eucalyptus* under different temperature, time, and solvent. **Fig. S1.** Schematic representation of Pd/C-catalyzed RCF of *Eucalyptus* sawdust. **Fig. S2.** Gas chromatograms and peak identification of the lignin monomers from catalytic hydrogenolysis of *Eucalyptus* sawdust using Pd/C catalyst. **Fig. S3.** ^1^H NMR spectra of 4-propanol syringol. **Fig. S4.** ^13^C NMR spectra of 4-propanol syringol. **Fig. S5.** ^1^H NMR spectra of 4-propanol guaiacol. **Fig. S6.** ^13^C NMR spectra of 4-propanol guaiacol. **Fig. S7.** The X-ray diffraction patterns of *Eucalyptus* sawdust and solid residue obtained from the RCF.

## Data Availability

All data generated or analyzed during this study are included in this published article and its supplementary information files.

## Abbreviations

5-HMF: 5-hydroxymethylfurfural
LA: levulinic acid
RCF: reductive catalytic fractionation
GVL: γ-valerolactone
M*w*: molecular weight
GPC: gel permeation chromatography
MWL: milled wood lignin
EMAL: enzymatic mild acidolysis lignin
MeTHF: 2-methyl tetrahydrofuran
DCM: dichloromethane
MIBK: methylisobutylketone
DMSO: dimethyl sulfoxide
