# Supplementary material for: Total utilization of lignin and carbohydrates in Eucalyptus grandis: an integrated biorefinery strategy towards phenolics, levulinic acid, and furfural
Source: Biotechnol Biofuels. 2020 Jan 6;13:2. doi: 10.1186/s13068-019-1644-z (PMC6943948; doi:10.1186/s13068-019-1644-z)
Supplement: Supplementary file 1 — Additional file 1: Table S1. The chemical composition of Eucalyptus sawdust and carbohydrate pulp. Table S2. Reductive catalytic fractionation of Eucalyptus under different temperature. Table S3. Reductive catalytic fractionation of Eucalyptus under different temperature, time, and solvent. Fig. S1. Schematic representation of Pd/C-catalyzed RCF of Eucalyptus sawdust. Fig. S2. Gas chromatograms and peak identification of the lignin monomers from catalytic hydrogenolysis of Eucalyptus sawdust using Pd/C catalyst. Fig. S3. 1H NMR spectra of 4-propanol syringol. Fig. S4. 13C NMR spectra of 4-propanol syringol. Fig. S5. 1H NMR spectra of 4-propanol guaiacol. Fig. S6. 13C NMR spectra of 4-propanol guaiacol. Fig. S7. The X-ray diffraction patterns of Eucalyptus sawdust and solid residue obtained from the RCF. [file 13068_2019_1644_MOESM1_ESM.pdf]

Additional file 1

Total Utilization of Lignin and Carbohydrates in *Eucalyptus grandis*: An Integrated Biorefinery Strategy towards Phenolics, Levulinic Acid, and Furfural

Xue Chen,[1] Kaili Zhang,[1] Ling-Ping Xiao,*[2] Run-Cang Sun,[2] and Guoyong Song*[1]

1. Beijing Key Laboratory of Lignocellulosic Chemistry, Beijing Forestry University, Beijing, 100083 (P.R. China)

2. Center for Lignocellulose Science and Engineering, Liaoning Key Laboratory of Pulp and Paper Engineering, School of Light Industry and Chemical Engineering, Dalian Polytechnic University, Dalian 116034 (P.R. China).

E-mail: songg@bjfu.edu.cn (G. Song); lpxiao@dlpu.edu.cn; lingpingxiao@163.com (L.-P. Xiao)

Contents

1	General Information	S2
2	The reductive catalytic fractionation (RCF) of *Eucalyptus* sawdust	S3
3	Chemical composition of *Eucalyptus* sawdust and carbohydrate pulp	S4
4	Lignin products analysis	S5
5	LA and FF analysis	S11
6	References	S13

1. General Information

GC and GC-MS: GC and GC-MS analyses were carried out on a Shimadzu Model 2010 plus equipped with a HP-5 column (30 m × 0.25 mm × 0.25 mm) using a flame ionization detector (FID) and a Shimadzu GCMS-QP2010SE equipped with a HP-5MS (30 m × 0.25 mm × 0.25 mm) column, respectively. The injection temperature was 250 ℃. The column temperature program was: 50 ℃ (3 min), 8 ℃/min to 280 ℃ (5 min). The detection temperature was 200 ℃ for FID.

GPC: GPC analyses were performed on Shimadzu LC20 series instrument via UV/vis spectroscopy at a wavelength of 254 nm, calibrated with polystyrene standards (peaks average molecular weights of 196, 580, 1320, 4830, 9970, Polymer Laboratories Ltd.). All lignin oil products were dissolved in THF (2 mg/mL) and filtered prior to injection.

NMR: The 2D-HSQC spectra of the lignin oil were determined on a Bruker AVIII 400 MHz spectrometer. About 40 mg of sample dissolved in 0.5 mL of DMSO-d_6 was used for determination. The main substructures in lignin oil were assigned according to previous reports [1, 2].

HPLC: LA was analyzed by HPLC (Shimadzu LC20 series) equipped with a reversed-phase C18-MS-II column (200 × 4.6 mm) and a refractive index (RI) detector. The column temperature was set at 40 ℃. The samples were eluted at 0.6 mL/min with acetonitrile and 0.1 wt% acetic acid aqueous solution (v:v = 15:85). FF was analyzed on a HPLC Aglilent-1200 system coupled with a UV detector (280 nm for detection wavelength). The column, elution requirement, and mobile phase were the same as LA test conditions.

2. The reductive catalytic fractionation (RCF) of *Eucalyptus* sawdust

General procedure: *Eucalyptus* (1.0 g), Pd/C (100 mg, 10 wt%) and methanol (40 mL) were mixed into a 50 mL stainless steel batch reactor (Parr Instruments Co.). The reactor was sealed, flushed with N_2 about three times and pressurized with 3 MPa H_2 at room temperature. The mixture was stirred at 800 rpm and heated at desired temperature. After reaction, the reactor was cooled to room temperature and depressurized. The reaction mixture was filtered through a nylon 66 membrane filter (0.2 μm), thus forming soluble (lignin oil) and insoluble fractions (carbohydrate pulp and catalyst). For the soluble fraction, the methanol was removed under vacuum, and the residue was extracted with dichloromethane and water to separate the degraded lignin (DCM phase) and sugar products (aqueous phase). The removal of dichloromethane of organic phase gave a brown "lignin oil", which was subjected to GC-MS and GC for analyze the lignin monomers.

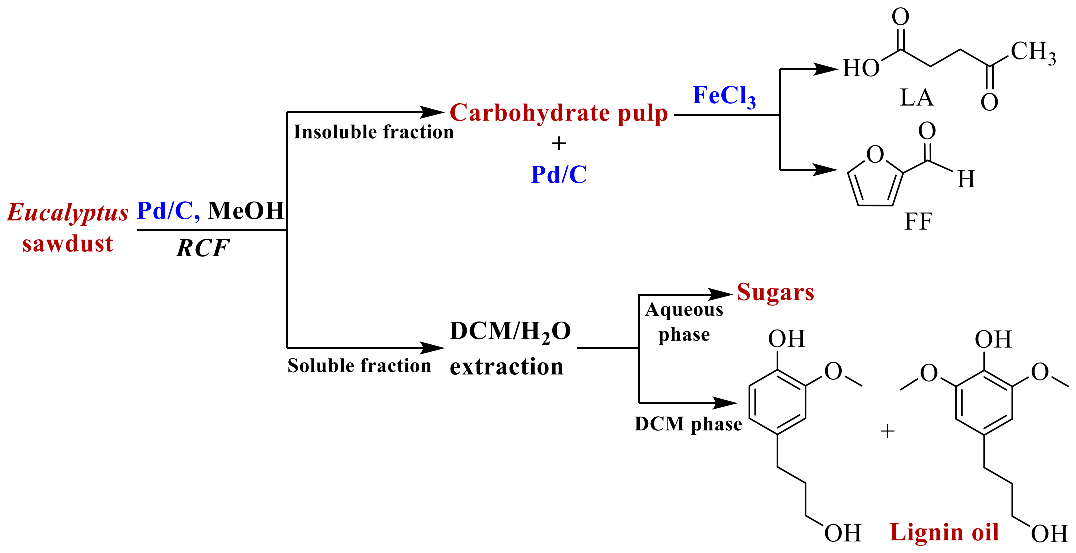

Fig. S1. Schematic representation of Pd/C-catalyzed RCF of *Eucalyptus* sawdust

3. Chemical composition of *Eucalyptus* sawdust and carbohydrate pulp

General procedure: The chemical composition was determined followed by National Renewable Energy laboratory's (NREL) standard analytical procedure. The sample was hydrolyzed at 30 ^{o}C with 72 wt% sulfuric acid solution (3.0 mL) for 1 h. Deionized water (84.0 mL) was then added to dilute sulfuric acid (ca. 3%). This mixture was heated at 120 ^{o}C for 1 h in the autoclave. After cooling, the mixture was filtered through a mixed cellulose ester (MCE) membrane filter (0.2 μm).

Analytical methods: The amount of acid insoluble lignin (AIL, Klason lignin) was determined by measurement the weight of residue after drying. The concentration of acid soluble lignin (ASL) was determined by UV spectra by measuring the absorbance of the soluble fraction at 205 nm. The determination of monomeric sugars in the aqueous soluble fraction was performed on high-performance anion exchange liquid chromatography (HPAEC) system (Dionex ICS 3000, USA) equipped with a Carbopac TM PA-20 column (3 mm × 150 mm, Dionex, Sunnyvale, USA) and an amperometric detector, by comparison with authentic samples. These samples were conducted in triplicate.

Table S1. The chemical composition of *Eucalyptus* sawdust and carbohydrate pulp[a]

Entry	Substrate	Temp.(^{o}C)	AIL[b] (wt%)	ASL[c] (wt%)	Cellulose (wt%)	Hemicellulose (wt%)
1	*Eucalyptus*	-	23.8	3.2	41.6	19.6
2	Carbohydrate pulp	200	13.5	2.4	50.5	25.6
3	Carbohydrate pulp	220	11.7	2.6	53.1	25.1
4	Carbohydrate pulp	230	10.6	2.5	57.1	23.9
5	Carbohydrate pulp	240	10.3	2.3	58.7	22.7
6	Carbohydrate pulp	250	10.7	2.5	49.0	19.2

[a] The chemical composition were analyzed according to the procedures of the NREL method.
[b] AIL: acid insoluble lignin (Klason lignin).
[c] ASL: acid soluble lignin.

4. Lignin products analysis

To analyze the lignin monomers after hydrogenolysis, the lignin oil and a standard (tetradecane) was solubilized in dichloromethane in a 10 mL volumetric flask. 10 μL was then analyzed on a GC (Shimadzu GC2010 series, equipped with a HP-5 column and a flame ionization detector (FID)) and GC-MS (Shimadzu GC2010 series equipped with a HP-5 MS column and a Mass Spectroscopy detector), respectively. The following operating conditions were used: injection temperature of 553 K, column heating program: 323 K (3 min), 10 K/min to 553 K, detection temperature of 563 K (for FID). The quantification of lignin monomers in the oil products were assessed by comparison with authentic samples acquired from commercial purchase or independent synthesis.

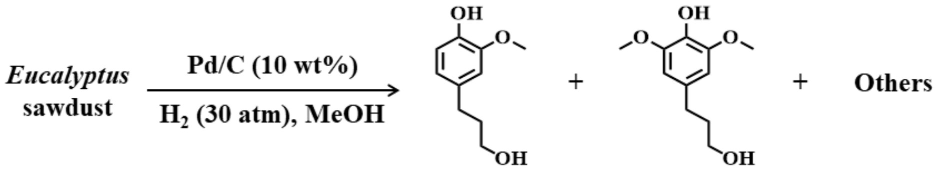

Table S2. Reductive catalytic fractionation of *Eucalyptus* under different temperature[a]

Entry	Temp. (^{o}C)	The distribution and yield of phenolic monomers (wt%) [b]							
		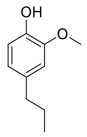	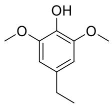	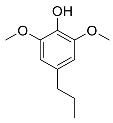	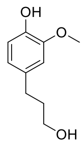	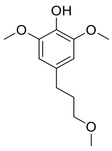	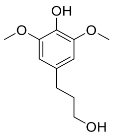	Others	Total
1	200	0.3	0.3	0.5	7.3	-	18.0	-	26.4
2	220	0.4	0.4	1.0	8.2	0.8	19.8	-	30.6
3	230	0.6	0.5	1.4	10.8	1.6	26.8	-	41.7
4	240	0.6	0.6	1.5	12.9	1.3	31.9	1.0	49.8
5	250	0.4	0.7	1.1	11.8	1.2	28.8	0.7	44.7

[a] Reaction conditions: *Eucalyptus* (1.0 g), 10 wt% Pd/C catalyst (100 mg), MeOH (40 mL), H_2 (30 atm), 4 h.

[b] Representing the monomer yield is based on lignin content in *Eucalyptus*.

Table S3. Reductive catalytic fractionation of *Eucalyptus* under different temperature, time, and solvent.

Entry	Solvent	Temp. (^{o}C)	Time (h)	Phenolic monomers yield (wt%)			
				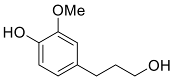	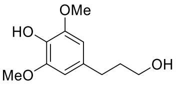	others	Total
1	MeOH	240	4	12.9	31.9	5.0	49.8
2	Ethanol	240	4	10.7	27.9	3.8	42.4
3	Isopropanol	240	4	9.6	26.8	3.6	40.0
4	H_2O	240	4	8.0	24.3	2.7	35.0
5	MeOH	200	8	9.1	23.2	4.3	36.6
6	MeOH	250	2	8.2	20.2	2.5	30.9

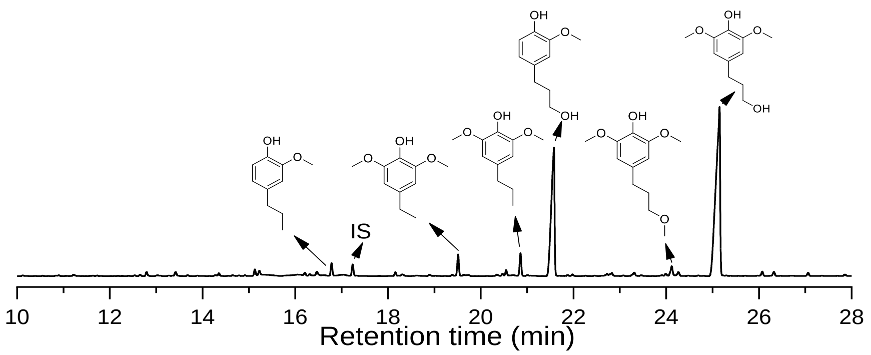

Fig. S2. Gas chromatograms and peak identification of the lignin monomers from catalytic hydrogenolysis of *Eucalyptus* sawdust using Pd/C catalyst. Reaction conditions: *Eucalyptus* (1.0 g), 10 wt% Pd/C catalyst (100 mg), MeOH (40 mL), H_2 (30 atm), 240 ^oC, 4 h.

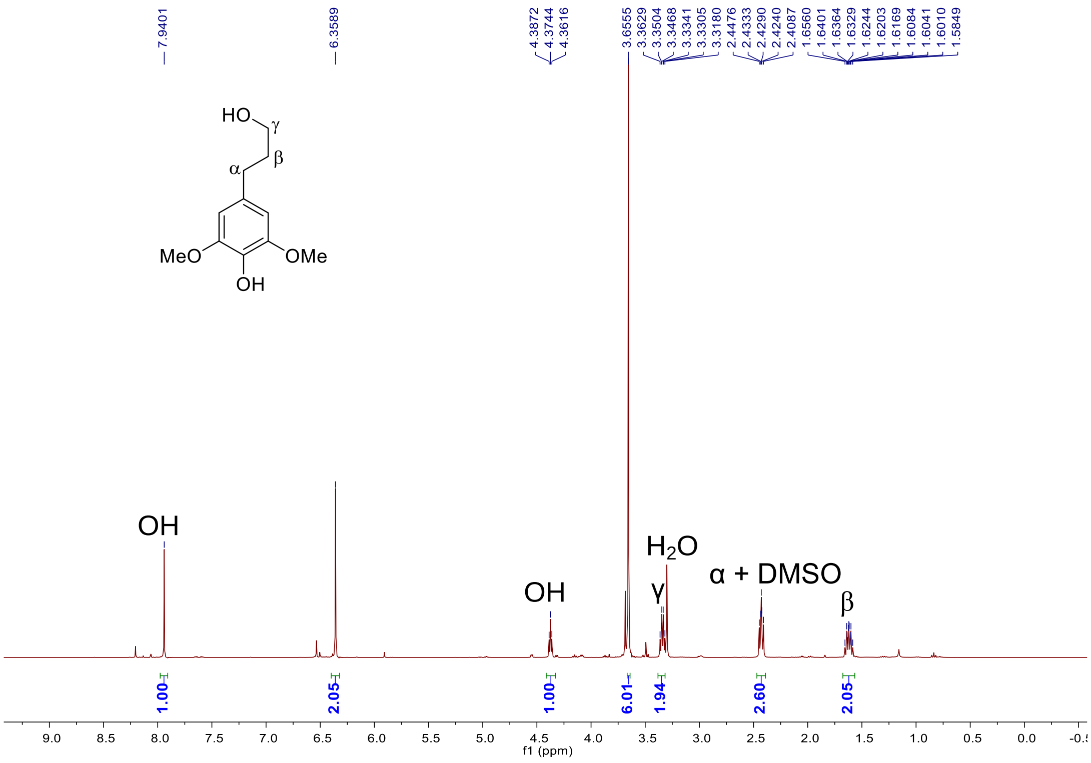

Fig. S3. ^{1}H NMR spectra of 4-propanol syringol.

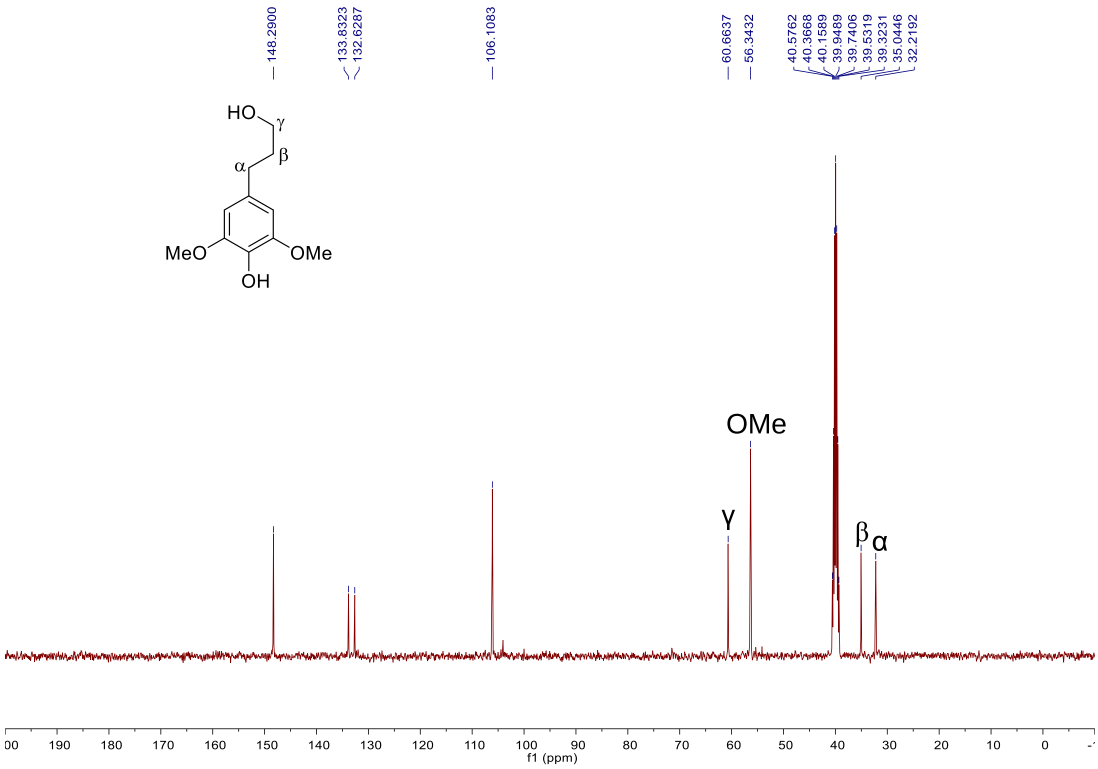

Fig. S4. ^{13}C NMR spectra of 4-propanol syringol.

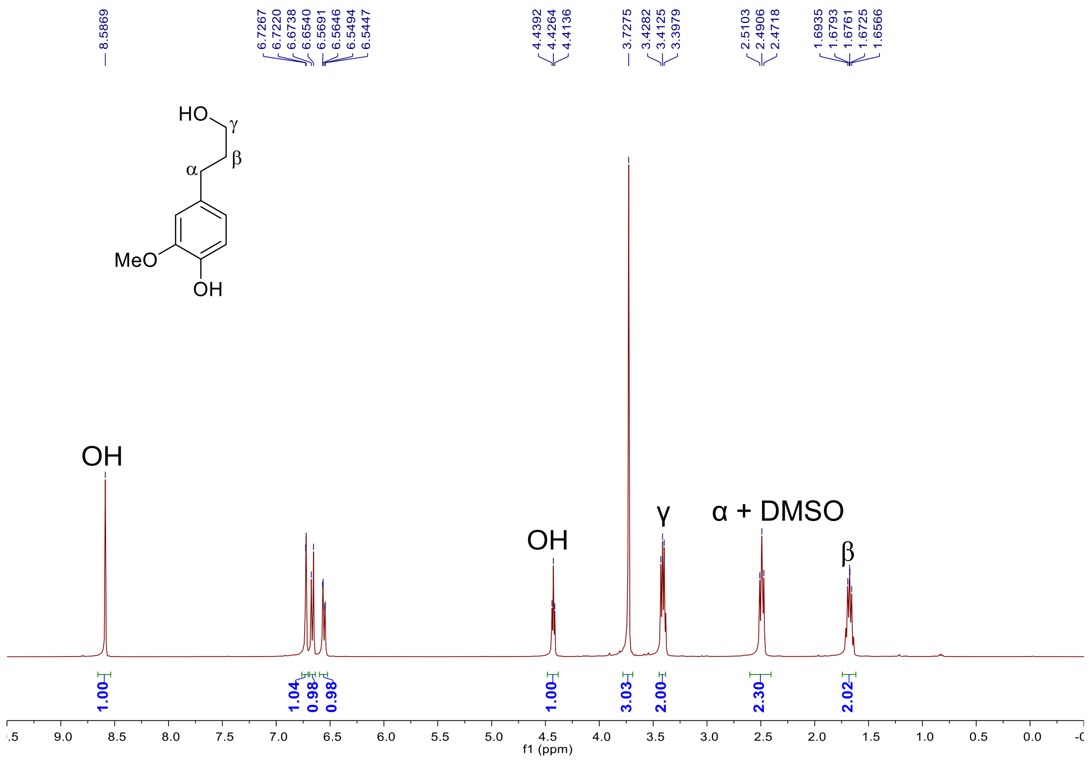

Fig. S5. ^{1}H NMR spectra of 4-propanol guaiacol.

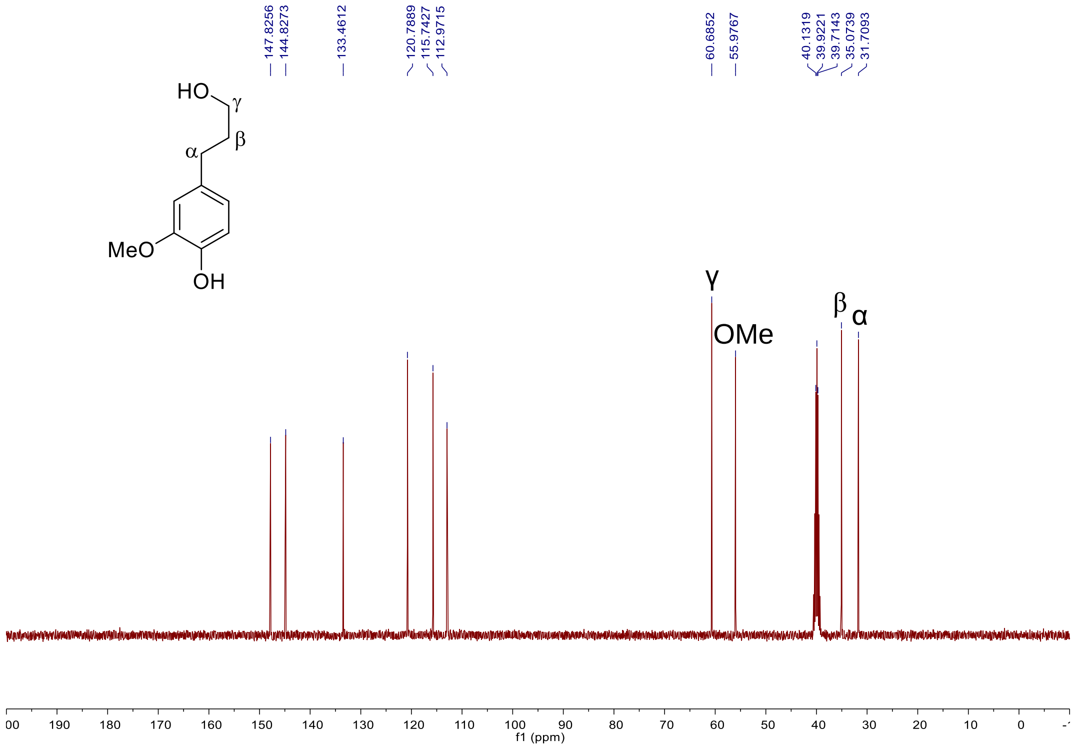

Fig. S6. ^{13}C NMR spectra of 4-propanol guaiacol.

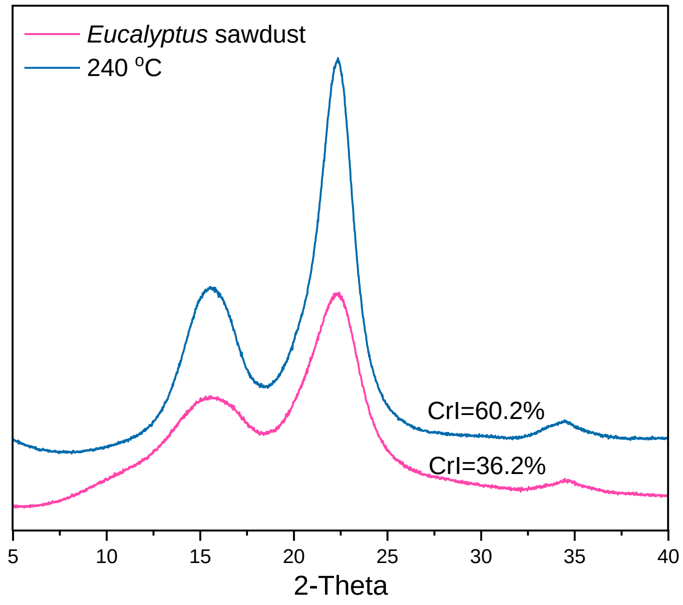

Fig. S7. The X-ray diffraction patterns of *Eucalyptus* sawdust and solid residue obtained from the RCF. Reaction conditions: *Eucalyptus* (1.0 g), 10 wt% Pd/C catalyst (100 mg), MeOH (40 mL), H_2 (30 atm), 240 ^oC, 4 h.

5. LA and FF analysis

LA analytical methods: LA was analyzed by HPLC (Shimadzu LC20 series) equipped with a reversed-phase C18-MS-II column (200 × 4.6 mm) and a refractive index (RI) detector. The column temperature was set at 40 ℃. The samples were eluted at 0.6 mL/min with acetonitrile and 0.1 wt% acetic acid aqueous solution (v:v = 15:85). The sample was diluted, and then 10 μL of the sample was injected for detection. The contents of LA in carbohydrate pulp were quantified by external standard calibration curve method as shown in Figure S9.

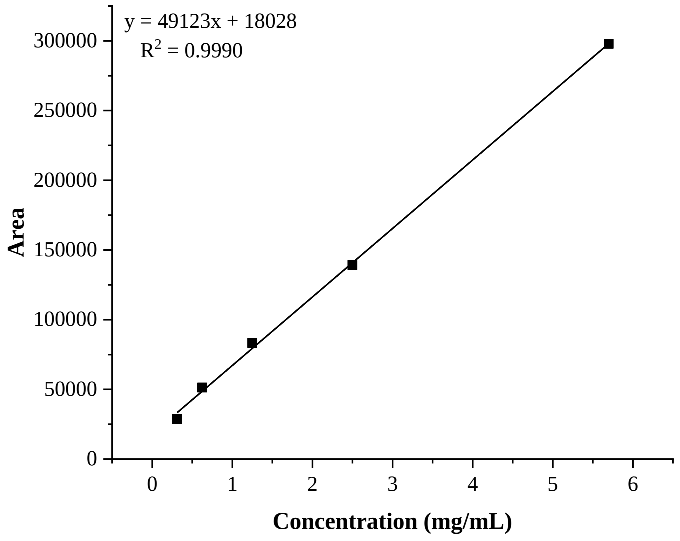

Fig. S8

FF analytical methods: FF was analyzed on a HPLC Aglilent-1200 system coupled with a UV detector (280 nm for detection wavelength). A reversed-phase C18-MS-II column (200 × 4.6 mm) with 40 ℃ column temperature was used for detection. The samples were eluted at 0.6 mL/min with acetonitrile and 0.1 wt% acetic acid aqueous solution (v:v = 15:85). The contents of FF in carbohydrate pulp were quantified by external standard calibration curve method as shown in Figure S10.

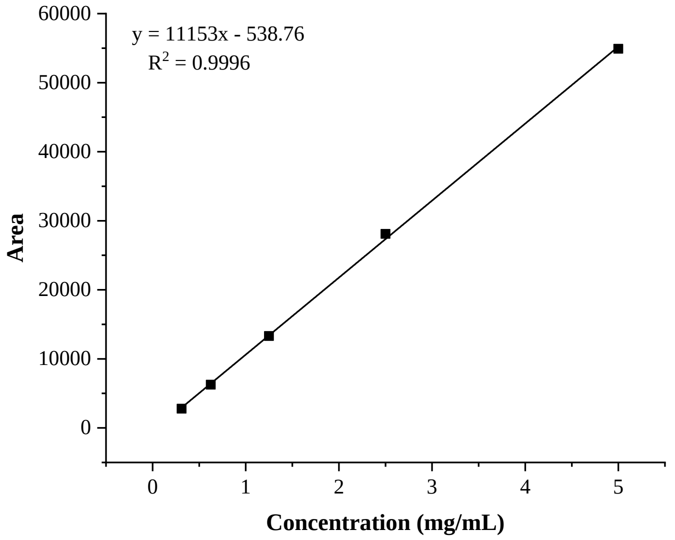

Fig. S9

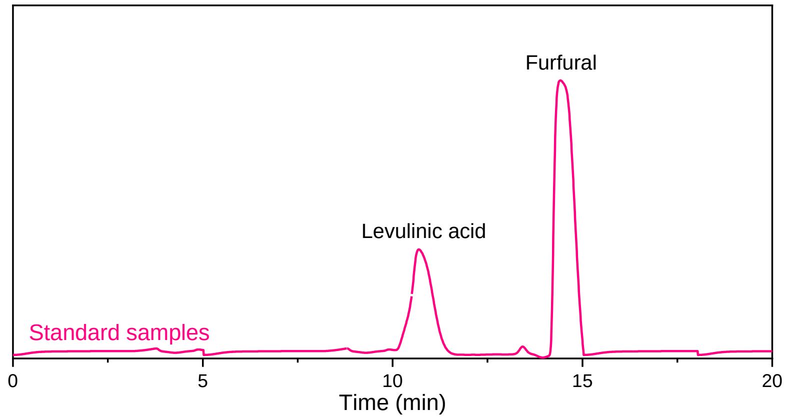

Fig. S10

6. References

1. Rencoret, J., et al., Lignin composition and structure in young versus adult Eucalyptus globulus plants. Plant Physiology, 2011. 155(2): p. 667-682.
2. Wen, J.-L., et al., Recent advances in characterization of lignin polymer by solution-state nuclear magnetic resonance (NMR) methodology. Materials, 2013. 6(1): p. 359-391.
